# Unravelling Transplant-Ineligible Newly Diagnosed Multiple Myeloma Treatment in Real-World Practice in Spain: The CARINAE Study

**DOI:** 10.3390/ph17101272

**Published:** 2024-09-26

**Authors:** Felipe de Arriba de la Fuente, Mercedes Gironella Mesa, Miguel Teodoro Hernández García, Juan Alonso Soler Campos, Susana Herráez Rodríguez, María José Moreno Belmonte, Teresa Regueiro López, Miriam González-Pardo, María Casanova Espinosa, on behalf of the CARINAE Study Investigators

**Affiliations:** 1 Hematology Department, Hospital Universitario Morales Meseguer, IMIB-Pascual Parrilla, Universidad de Murcia, 30008 Murcia, Spain; farriba@um.es; 2 Hematology Department, Hospital Universitario Vall d’Hebrón, 08035 Barcelona, Spain; mgironel@vhebron.net; 3 Hematology Department, Hospital Universitario de Canarias, 38320 La Laguna, Spain; mthernan@ull.edu.es; 4 Hematology Department, Hosptial Universitario Parc Taulí, 08208 Barcelona, Spain; jsoler@tauli.cat; 5 Hematology Department, Hospital Universitario Basurto, 48013 Bilbo, Spain; susana.herraezrodriguez@osakidetza.eus; 6 Hematology Department, Hospital Universitario Virgen de la Arrixaca, 30120 Murcia, Spain; mjmobel9@hotmail.com; 7 Spanish Multiple Myeloma Patient Community (CEMMP), 24560 León, Spain; tregueiro@comunidadmielomamultiple.com; 8 Medical Department, Janssen-Cilag S.A., Johnson & Johnson Company, 28042 Madrid, Spain; 9 Department of Hematology/Clinical Oncology, Hospital Costa del Sol, 29603 Marbella, Spain; mariacasanova@yahoo.com

**Keywords:** multiple myeloma, monoclonal antibodies, immunotherapy, chemotherapy, daratumumab

## Abstract

Real-world evidence on the impact of monoclonal antibodies as first-line treatment in Spain is limited. This observational, retrospective and prospective, multicenter, descriptive study included 117 transplant-ineligible newly diagnosed multiple myeloma (TIE-NDMM) patients divided into Group A, who received no daratumumab standard regimens, and the DVMP group (daratumumab, bortezomib, melphalan, and prednisone treatment). More than 90% of the patients in Group A received bortezomib, lenalidomide, or a combination of them. The median follow-up time for Group A was 38.2 months in comparison to 25.8 months for the DVMP group (*p* < 0.0001). The rate of DVMP patients that experienced disease progression or death from any cause was 36.8%, compared to 67.3% of Group A patients at 36 months of follow-up. The DVMP group had a higher 36-month progression-free survival (PFS) rate (52.9% vs. 31.7%). During the retrospective period, 73.0% of patients reported adverse drug reactions, while in the prospective period, 40.5% experienced adverse events, with no clinical differences between groups. The study supports the use of daratumumab regimens in frontline therapy based on real-world data. The findings provide valuable insights into the clinical outcomes of daratumumab therapy, which can help physicians make informed decisions regarding the optimal treatment approach for this patient population.

## 1. Introduction

Multiple myeloma (MM) accounts for 10% of all hematological malignancies [1] and is responsible for 1.8% of all cancer-related deaths in Spain [2]. This disease is still considered incurable, and relapse or disease progression after first-line treatment is common. Most patients are diagnosed after the age of 65, which remains a significant challenge because this group has comorbidities such as kidney failure, peripheral neuropathy, cardiovascular disease, and nutritional deficiency, and is not eligible for transplant. Therefore, treatment consists of a combination of systemic chemotherapy and immunomodulatory agents, leading to a balance between effectiveness and toxicity [3,4,5]. Another prognostic factor is the presence or absence of cytogenetic high-risk abnormalities such as del (17 p), t (4;14), t (14;16), t (14;20), 1 q, p53 mutation, gain (1 q), or del (1 p) [2,6,7]. Up to 40% of patients with multiple myeloma do not qualify for phase III clinical trials; therefore, it is unclear how to best treat these patients. It is undeniable that real-world data significantly complement the results of clinical trials, making this type of analysis increasingly common. Real World Evidence (RWE) studies provide essential information for healthcare professionals to optimize treatment, improve patient outcomes, and deliver more efficient healthcare. This includes effectiveness and tolerability, quality of life, economic impact, treatment satisfaction, and patient preference [8,9,10].

Daratumumab is the first approved anti-CD38 monoclonal antibody with direct anti-tumor and immunomodulatory activity. Daratumumab induces rapid, deep, and durable clinical responses in patients with MM through a multifaceted mechanism of action. Direct on-tumor actions include complement-dependent cytotoxicity, antibody-dependent cellular cytotoxicity, antibody-dependent cellular phagocytosis, and induction of apoptosis by cross-linking [11,12]. Daratumumab is also considered active immunotherapy due to its immunomodulatory effects. It exerts immunomodulatory effects via T-cell induction/expansion, T-cell activity enhancement, and reduction of immune-suppressive cell populations [13]. This activity may contribute to prolonged and deep clinical responses [14].

Daratumumab has shown benefit in newly diagnosed and relapsed/refractory MM patients by improving progressive free survival (PFS) and overall survival (OS) with manageable side effects in TIE-NDMM patients. In the ALCYONE clinical trial, Daratumumab was combined with Bortezomib, Melphalan, and Prednisone (VMP); and in the MAIA clinical trial with Lenalidomide and Dexamethasone (Rd). Both were multicenter, randomized, open-label, active-controlled trials with approximately 700 patients each, demonstrating a higher PFS and OS in combination with Daratumumab. The ALCYONE trial showed a 36-month rate PFS of 50.7% in the DVMP group versus 18.5% in the control group (VMP), while the MAIA trial demonstrated a 30-month rate PFS of 70.6% in the D-Rd group versus 55.6% in the control group (Rd) [15,16,17]. Non-transplant eligible patients have experienced improved quality of life [18,19] and prolonged PFS with first-line regimens based on daratumumab. Remarkably, their outcomes are approaching those of transplant-eligible patients treated with triplet therapies. This is a significant emotional milestone for these patients, as it brings their prognosis closer to that of younger patients [20,21]. The 2021 EHA-ESMO guidelines recommended these combinations as a first-line treatment because they were more effective than standard therapies [22]. The efficacy and safety data of daratumumab, as well as its recommendation by therapeutic guidelines, have led to more than 518,000 patients currently being treated with daratumumab worldwide [23]. However, there is limited evidence of daratumumab’s effectiveness and the impact of its use on the outcomes of TIE-NDMM patients in Spain outside of clinical trials.

In addition to clinical factors, patients’ perspectives of the disease’s burden and the therapy’s impact on daily life should be considered. Within the patient community, a greater need for knowledge of the disease itself and its treatments is identified, providing a new perspective that must be taken into consideration by the scientific community.

CARINAE is an observational study that describes the impact of daratumumab treatment in front-line therapy versus other first-line treatments without the monoclonal antibody in daily clinical practice in Spanish hospitals from 2020 to 2023.

## 2. Results

### 2.1. Patients and Treatment

Thirty Spanish hospitals included 117 patients after an 11-month recruitment period between December 2020 and October 2021. Patients continued with prospective follow-up for 18 months, until April 2023. Patients who did not receive daratumumab treatment are classified as Group A, as opposed to those who received DVMP therapy. For group A, first-line treatment started between September 2018 and August 2019, while for the DVMP group, it began between September 2019 and November 2020. Patients treated with DRd were not included, as at that time it was not yet reimbursed in the Spanish national health system, and therefore not widely available. In March 2020, Spain declared the SARS-CoV-19 pandemic, which had a more significant impact on the DVMP patient group during the initial treatment and prospective follow-up.

A total of 109 patients were evaluated for effectiveness, with 52 in Group A and 57 in the DVMP Group. There were 111 patients assessed for safety, with 53 in Group A and 58 in the DVMP Group. Since 16 patients were deceased before starting the study (11 in Group A and 5 in the DVMP Group), with only retrospective data available, 93 patients remained in the follow-up period.

Patients’ demographic and clinical characteristics were similar between the two groups, with 54.1% men, a mean (SD) age at diagnosis of 75.8 (5.3) years, and 21.1% aged 80 years or older. The mean (SD) weight was 72.0 (13.4) kg, and the mean (SD) Body Mass Index (BMI) was 27.6 (4.9) kg/m^2^ (Table 1).

Relevant comorbidities included heart disease in 28.4% of patients and kidney failure in 24.8% of patients. Significant differences were only observed in moderate/severe chronic kidney failure, 19.2% in Group A vs. 3.5% in the DVMP group. Most patients (82.5%) had an ECOG performance status between 0 and 2 at the time of diagnosis, 61.1% of patients had documented lytic bone lesions, and 24.1% had plasmacytomas (Table 1).

Patients in both groups had similar ISS and R-ISS stages. Approximately half of the patients were classified as being in R-ISS stage II, 17.4% in stage I, and 15.6% in stage III. In the ISS classification, patients were similarly distributed (Table 1).

The most common first-line treatment for patients in group A was a Bortezomib-based scheme, with 32.7% undergoing VMP treatment and only 5.8% receiving Bortezomib-Lenalidomide-dexamethasone. Lenalidomide-dexamethasone was administered to 26.9% of patients, and melphalan-prednisone was given to 3.8% (Table 2).

First-line treatment was administered until progression to most patients in Group A (75.0%) and all patients in the DVMP Group (100.0%), with no significant differences between groups in the number of cycles received (Table S2). The shorter follow-up time for DVMP patients must be considered to interpret these results.

### 2.2. Laboratory/Molecular Findings

The patient’s initial evaluation showed similar hemoglobin levels in both groups, with a mean (SD) of 11.2 (2.2) g/dL and a range between 6.5 g/dL and 16.4 g/dL, and no cytopenia was reported. Calcium, albumin, and β−2 microglobulin levels were similar between groups, with mean (SD) values of 9.6 (0.9) mg/dL, 3.7 (0.6) g/dL, and 6.3 (5.6) mg/L, respectively. Approximately 13.2% of patients exhibited elevated LDH levels. Kidney impairment (ClCr ≤ 60 mL/min) was observed in 29.9% of patients, with a creatinine clearance of less than 30 mL/min in 11.9%. The quantification of M protein in serum and urine was high and similar in both groups, with mean (SD) values of 2751.0 (2901.3) mg/dL and 662.8 (1053.4) mg/24 h. Serum immunofixation was positive in 62.4% of patients, although the information was not available in 33.0% of cases. Both groups had high levels of kappa and lambda FLC in serum (median (P25–P75) 18.0 (1.3–81.6) mg/dl and 2.6 (0.7–36.1) mg/dl) and the FLC κ and λ ratio (median (P25–P75) 6.5 (0.0–60.0)) (Table S1).

The bone marrow analysis revealed a mean (SD) of 34.4% (21.8) of clonal plasma cells; however, no patient had circulating plasma cells in the peripheral blood.

Nearly half of the patients in both groups had cytogenetic alterations, with 24% having a high cytogenetic risk [del (17 p), t (4;14) and t (14;16)]. Extended high-risk cytogenetic alterations (including gain or amplification of 1 q21) were observed in 66% of patients.

### 2.3. Effectiveness. Primary Objective

**Figure 1:**
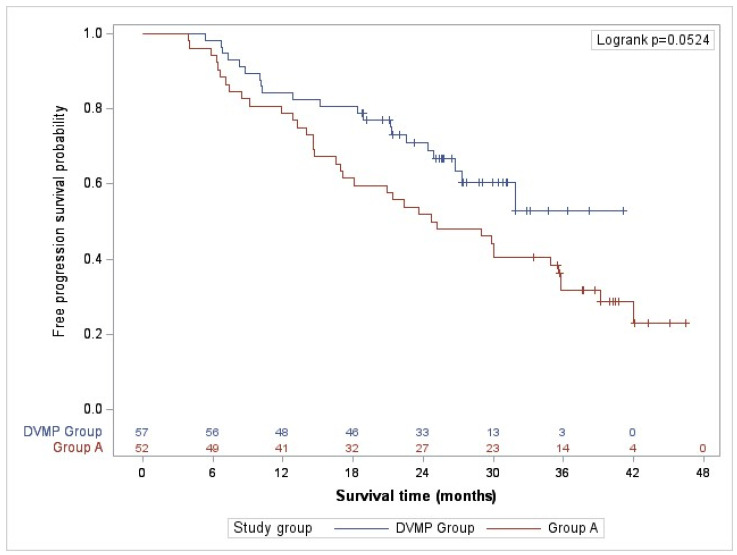
Progression-free survival (PFS).

The study’s primary objective was to describe the progression-free survival (PFS) of daratumumab in combination with VMP, and of the alternative treatment regimens. The median progression-free survival for group A was 24.97 months (95% CI, 16.90–35.57), with a median follow-up of 38.2 months (Table 3). The median PFS for the DVMP group could not be estimated because the survival probability at the end of the follow-up was >0.5, with a median follow-up of 25.8 months. The rate of DVMP patients who experienced disease progression or death from any cause was 19.3% compared to 38.5% of Group A patients at 18 months of follow-up, 28.1% vs. 48.1% at 24 months and 36.8% vs. 67.3% at 36 months, observing consistent differences at the end of the study (36.8 vs. 71.2%). Additionally, patients treated with DVMP had a higher likelihood of survival (0.5285 versus 0.2306). The 36-month progression-free survival rate was 52.9% in the DVMP group and 31.7% in group A (Table 3 and Figure 1).

PFS censored by other death–disease progression or death due to progression was 57.7% of patients in Group A and 24.6% in Group DVMP (Figure S1).

The median PFS for patients over 80 years of age (23 patients) in the complete follow-up was 28.39 months (95% CI, 7.31-NA) in the DVMP group vs. 18.91 months (95% CI, 5.88–28.93) in group A. However, no statistically significant differences were observed in survival probability.

### 2.4. Clinical Response to Therapy

First response assessment was performed after a mean (SD) of 61.0 (74.8) days and a median of 42.0 days after the initiation of first-line treatment, with no differences between groups. In the first evaluation, 28.4% of patients achieved a very good partial response or better (≥VGPR), with significant differences observed between patients in the group treated with DVMP (36.8%) compared to Group A (19.2%) (*p* = 0.0418).

Regarding the best response, 88.1% of patients achieved partial response or higher with first-line treatment in a mean (SD) of 70.8 (81.9) days, with no differences observed between study groups (82.7% group A vs. 93% DVMP), and a mean (SD) response duration of 1.9 (0.9) years. The best response was a complete response or better in 36.8% of patients in the DVMP group vs. 28.8% in group A (*p* = 0.3753), and a very good partial response or better in 75.4% of the DVMP group vs. 57.7% in group A (*p*= 0.0491) (Table 4).

Only 20 patients (18.3%) underwent minimal residual disease evaluation. The results were positive in 47.6% of patients and negative in 52.4%. We found no statistically significant differences between the treatment groups.

The percentage of patients with disease progression after starting first-line treatment was significantly higher in the alternative treatment group than in the DVMP group (57.7% vs. 24.6%), as well as the patients who had disease progression after achieving PR or better (58.1% vs. 22.6%). Different follow-up times between groups must be considered to interpret these results. Relapse characteristics were similar in both groups; 45.5% of the relapses to first-line treatment were symptomatic, 29.5% were biological with criteria to start treatment, and 25.0% were without it (Table 5).

### 2.5. Overall Survival

**Figure 2:**
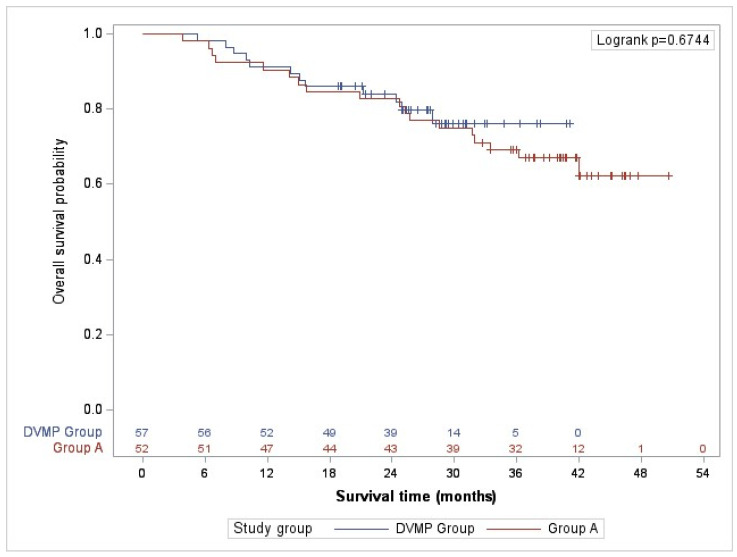
Overall survival.

The study revealed that patients who received DVMP treatment had a mortality rate at 36 months of follow-up of 21.1%, whereas those in the alternative treatment group had a mortality rate of 30.8%. Results were consistent at complete follow-up (21.1% vs. 34.6%). Although the DVMP treatment demonstrated superior overall survival probability, the differences were not statistically significant during the study (Figure 2).

### 2.6. Treatment Management in Clinical Practice in Spain

Treatment was prescribed until progression in 75% of patients in Group A (Table S2). Among them, 76.9% discontinued the treatment after a mean (SD) of 384.4 (343.4) days, principally by medical decision (53.3%) and treatment-related adverse events (40.0%). Of the remaining 25% of patients who received fixed treatment, 92.3% finalized or interrupted treatment after a mean (SD) of 335.5 (123.8) days, mainly due to treatment completion (58.3%) and medical decision (25.0%).

In the DVMP group, treatment was prescribed until progression (Table S2). Among them, 36.8% ended treatment during the study, primarily due to medical decision (53.3%) (52.4%), death (23.8%), and treatment-related adverse events (19.0%).

During follow-up, Group A patients received significantly more subsequent, second line and beyond, multiple myeloma treatments (59.6%) than Group DVMP (22.8%); in total, 19 of them were treated with anti-CD38, 15 patients (48.4%) in Group A and 4 patients (30.8%) in Group B (Table S3).

No significant differences were observed between groups in the time to the next anti-myeloma treatment after progression of first-line treatment, with a mean (SD) of 2.1 (1.0) years (Table S3). It is important to consider the shorter follow-up time for the DVMP when interpreting this result.

### 2.7. Safety

During retrospective follow-up, 73.0% of patients (*n* = 81) reported 251 adverse reactions to medication (RAMs), of which 11.2% were serious (Table 6). Hospitalization was the most frequent severity criterion (50.0%), and the most commonly reported RAMs were blood and lymphatic disorders (41.8%), followed by nervous system disorders (13.1%) and gastrointestinal disorders (12.0%). The study analysis showed no significant differences between groups regarding the proportion of patients who experienced adverse reactions or their severity, despite differences in their intensity.

During the prospective follow-up, 40.5% of patients (*n* = 45) reported 172 adverse events (AE) in first-line treatment, of which 15.1% were serious (Table 7). The most frequent severity criterion was hospitalization (76.9%). Infections (25.6%) were the most frequent events, followed by gastrointestinal disorders (13.4%) and nervous system disorders (11.0%). Infections (46.2%) were the most common serious adverse events (SAEs), followed by cardiac disorders (15.4%). We observed statistical differences between groups in terms of the proportion of patients with adverse events and severity. However, these differences were not clinically relevant if we consider that the Group DVMP has a significantly higher proportion of patients on first-line treatment during prospective follow-up than the alternative group (75.4% vs. 28.8%).

Among the patients aged 80 and older (*n* = 25 in the safety sample), 18 individuals had 57 RAMs in the retrospective period, 14.0% serious, and 8 patients had 21 AEs in the prospective period, 14.3% serious. There were no significant differences between the two groups.

### 2.8. Patient’s Study Participation and Perspective

To differentially incorporate the patient’s voice into the CARINAE study, two main aspects were addressed: (1) direct collaboration with the Spanish Multiple Myeloma Patient Community (CEMMP) in the creation and implementation of the study (review of the protocol, case report form and patient information documents, as well as design of a questionnaire on different aspects of the disease from the patient’s perspective) and (2) understanding, through these questions, the patient’s impression of the disease perception, quality of life, and treatment adherence.

A total of 66 patients (30 from Group A and 36 from Group DVMP) answered a questionnaire to assess the patient’s perspective 24 months (±4 weeks) after the start of first-line treatment (Figure S2). Most patients expressed concern about the disease, mainly in terms of feeling worse. However, most patients looked to the future with optimism, felt capable of enjoying life, were satisfied with the available disease information, and had a better perception of their health post-MM treatment. Some patients needed to share their experiences and concerns with others. More than half of the patients consistently or frequently reported difficulties with strenuous activities or long walks, but not with daily tasks or short walks. Regarding symptom improvement, most patients experienced relief or disappearance of pain since their diagnosis. When evaluating a treatment, patients identified efficacy as the most important factor, while the route of administration was the least important. Over 90% of patients were satisfied with symptom control, with good coexistence with treatment, and reported good adherence to treatment and disease follow-up.

## 3. Discussion

The baseline age of patients included in this observational study, conducted in the context of daily clinical practice in Spain, was similar to those published in randomized trials. The ALCYONE trial [15,16] randomized 706 TIE-NDMM patients of a mean age of 71 years old into two groups (DVMP vs. VMP), the VISTA trial [24,25] randomized 682 MM patients ineligible for high-dose therapy and a mean age of 71 years old into two groups (VMP vs. MP), and the FIRST trial [26] randomized 1623 TIE-NDMM patients of a mean age of 73 years old into three groups (continuous lenalidomide–dexamethasone, 18 cycles of lenalidomide–dexamethasone, or Melphalan-Prednisone-Thalidomide). However, our study included real-world patients with more heterogeneous ECOG scores, including ECOG 3 and 4, as well as comorbidities such as cardiovascular, lung, and kidney diseases.

Despite including patients with more variability, the effectiveness results observed in this study were consistent with efficacy results obtained under controlled conditions in clinical trials. PFS was higher in the DVMP group than in the alternative treatment regimens group, consistent with the results obtained in the ALCYONE study with a 36-month PFS of 50.7% in the Daratumumab group.

The alternative treatment group had a significantly higher percentage of patients with a first-line treatment disease progression than the DVMP group (57.7% vs. 24.6%). Different follow-up times between groups must be considered to interpret these results, but superior PFS at 18, 24, and 36 months in favor of DVMP supports this trend. Likewise, in the ALCYONE clinical trial, disease progression was more significant in the control group, 65%, compared to 41% in the daratumumab group [15,16].

Although the overall survival (OS) probability in the DVMP Group was higher throughout the study, the differences were not significant. The results were consistent with clinical trials. Overall survival at 36 months in the ALCYONE clinical trial was 78% in the experimental group against 67% in the control group [15,16]. In the VISTA clinical trial, the three-year OS was 68.5% versus 54% in the group without bortezomib. In the FIRST clinical trial, the four-year OS was 59% in the first experimental group compared to 51% in the control group [24,25,26]. It should be noted that a higher number of patients in group A received subsequent treatments that included antiCD38 (daratumumab or isatuximab), which may have contributed to the lack of significant differentiation between the two groups in the overall survival curve.

The best response observed in the CARINAE study, which was superior in the DVMP Group, was also comparable to the ALCYONE clinical trial, with 73% of patients having a very good partial response or better and 46% of patients having a complete response [15,16]. The effectiveness results in group A were consistent with those obtained, for example, in the VISTA clinical trial [24,25]. With a follow-up of 36.7 months, the group treated with the VMP regimen had a 35% lower risk of death compared to the group without bortezomib. These results also align with those from the FIRST clinical trial [26]; with a mean PFS of 25.5 months versus 21.2 months in the MPT group. It should be noted that in the context of daily clinical practice, it was observed that the time to the first response assessment, while recommended after the first treatment cycle, averaged more than two months in this study.

Achieving MRD negativity is particularly important in high-risk patients and is crucial to achieving long-term outcomes [6]. However, only 20 patients (18.3%) underwent minimal residual disease evaluation. The Spanish Myeloma Group (GEM) is one of the cooperative groups that has contributed the most to generating the scientific evidence that supports the relevance of negative MRD in MM, placing it as a desirable treatment goal [27,28]. GEM has also reported preliminary results from a study evaluating MRD in RW practice in Spain [29], which could complement the results of the present study. To facilitate wider access to MRD RW measurements in Spanish hospitals, the Cavex program endorsed by GEM, makes it available through its analysis by high-sensitivity multidimensional flow cytometry through a national network of three internationally validated reference sites. Since its launch in 2015, 137 hospitals have joined, and 5440 samples have been analyzed to date [30].

Combining daratumumab with bortezomib, melphalan, and prednisone did not increase toxicity, and adverse events were evenly distributed between the DVMP group and group A. During the retrospective period of the study, no differences were observed between groups in terms of the proportion of adverse drug reactions or severity. During the prospective period, differences between groups were observed both in the proportion of adverse events and their severity, but they were of little clinical relevance considering that the D-VMP group had a significantly higher proportion of patients on first-line treatment during the prospective follow-up than the alternative treatment group (75.4% vs. 28.8%).

The group of MM patients older than 80 years remains a relevant challenge, since this group accumulates more comorbidities, frailty, disability and is frequently underrepresented in clinical trials. In a recent publication focusing on patients over 80 years of age, treated in real clinical practice with a doublet bortezomib-based regimen, the results showed, after a median follow-up of 31.4 months, a median PFS and OS of 13.2 and 26.9 months, respectively, with an invitation for reflection on ways to improve management in a super elderly/very frail patient population [31]. Although the representation of the over−80 s in our study is small, a trend can be identified in the improvement of PFS (28.39 months (7.31-NA) vs. 18.91 months (5.88–28.93) in group A, differences were not statistically significant) with the addition of immunotherapy in the first line in the absence of a significant increase in toxicity, ultimately providing an improved treatment tool to be considered in these older patients.

We are entering a stage where patients will increasingly align with other medical professionals to actively participate in the development of their disease management and treatment. Patient involvement in clinical trials is essential for their successful execution. In recent years, patient associations, including the Spanish Community of Multiple Myeloma Patients (CEMMP), have demonstrated the benefit of including the patient perspective. This goes beyond the recruitment phase, nurturing the design, development, and execution of clinical trials. With more treatment options available, it is crucial to understand patient preferences when making decisions to provide the best possible treatment for patients with multiple myeloma [32]. Our study seeks to increase awareness about the patient burden and the impact of an incurable multiple myeloma diagnosis on their life. The study facilitated the participation of patients not only as participating subjects but also as an enriching figure in the implementation of a study of these characteristics in our country. The CARINAE study aimed to gain insight into patients’ opinions on the information provided by their attending physicians, their understanding of the disease, the impact of treatment on their daily life, and how they balance life and treatment.

## 4. Materials and Methods

### 4.1. Study Design and Patient Population

This is an observational, retrospective and prospective, multicenter study to describe the repercussions of monoclonal antibodies as first-line treatment among other standard regimens in TIE-NDMM patients in daily clinical practice in thirty Spanish hospitals. The study had a recruitment period from 1 December 2020 to 28 October 2021, with a prospective follow-up period from December 2020 until April 2023.

Patients with newly diagnosed, documented multiple myeloma who were not eligible for autologous stem-cell transplantation because of coexisting conditions or older than 65 years were recruited consecutively. All live patients provided written informed consent before being included in the study.

We excluded patients with primary amyloidosis, monoclonal gammopathy of undetermined significance, smoldering multiple myeloma, Waldenström’s macroglobulinemia (or other conditions characterized by the presence of IgM paraprotein in the absence of clonal plasma cell infiltration with lytic bone lesions), and prior systemic therapy or stem cell transplant.

The patients in group A started treatment twelve months before the reimbursement of daratumumab-VMP and were given a combination of at least two drugs without Daratumumab (September de 2018–August 2019). Patients in the DVMP group started first-line treatment within 15 months of the reimbursement of this combination in the Spanish national health system (September 2019–November 2020). Data was collected from the source documents (each participating patient’s medical records) and entered in a designed electronic case report form (eCRF).

The effectiveness and safety populations included all patients who met all inclusion criteria and none of the exclusion criteria, received at least one dosage of the study treatment, and had at least one response evaluation.

### 4.2. Primary and Secondary Endpoints

The study’s primary endpoint was progression-free survival (PFS) in each treatment arm, defined as the number of months between the beginning of the first-line treatment and the date of disease progression or death from any cause, whichever occurred first. Time-to-event variables, such as progression-free survival, were evaluated using the Kaplan–Meier method. The log-rank test was used to do statistical comparisons of survival time across treatment groups (A or DVMP).

The clinical response rate has been described for the total sample and each treatment group (A or DVMP), considering the first evaluation of the response after the start of treatment, the best response during treatment, the size or lack of partial response (PR), and progression or absence of the disease (PD). The duration of the response, defined as the time in months between the first observation of response (PR or better) and the first evidence of PD or death due to progression, has been described and compared between groups.

Overall survival was defined as the time in months between the date of initiation of first-line treatment and the date of death from any cause. The analysis of overall survival was performed using the Kaplan-Meier method, and the median with a 95% confidence interval was provided. Statistical comparisons of survival time by treatment group were made using the log-rank test.

Safety assessments included graded evaluation of adverse events following the NCI CTCAE (version 5.0 of the NCI). Verbatim terms used in the eCRF by the participating physician to document adverse events have been coded using the MedDRA dictionary, version 26.0. All documented adverse events were included in the analysis.

### 4.3. Statistical Methodology

This study does not aim to confirm/reject any pre-established hypothesis. The statistical analyses are purely descriptive. Categorical variables were described using absolute and relative frequencies. To describe the continuous variables, we used the mean, the standard deviation (SD), the 95% confidence interval (CI) for the mean, the median, the 25th (P25) and 75th (P75) percentiles, and the minimum (Min) and maximum (Max), including the total number of valid values.

All the variables collected in the eCRF were described for the total sample and each treatment group (Group A or DVMP Group), and statistical comparisons between groups were made using Student’s *t*-tests or Mann-Whitney U tests on quantitative variables or through Chi-square tests or Fisher’s exact test in qualitative variables, depending on the characteristics of the variables under study (type and normality).

Progression-free survival (PFS) and overall survival (OS) were estimated using the Kaplan-Meier method, presenting the median with a 95% confidence interval and the corresponding survival graph. All statistical comparisons were performed using a two-sided test with a significance threshold of 0.05. The statistical treatment of the data was carried out with the support of the SAS statistical package version 9.4.

### 4.4. Limitations

One of the study’s limitations is its retrospective nature (part of it). In retrospective studies, the data is obtained from the patient’s medical records and may be unavailable or incomplete. Others are inherent to its observational and nonrandomized design (including the possibility of residual confounding due to unobserved treatment selection biases). Selection bias in patient enrolment cannot be discarded either. Trying to avoid this, consecutive recruitment of patients in the sites has been carried out and representative sites have been selected from the entire Spanish territory.

The data should be interpreted with caution, considering the significant variability in the treatment regimens of Group A. In many cases, clinical and treatment management does not follow established protocols, or are adapted to the peculiarities of the hospital or the patient. Due to these same limitations, these types of studies are essential for complementing data obtained in controlled clinical trials regarding effectiveness or safety and for knowing the real patients’ characteristics and behavior.

The aggregate analysis in only one treatment group (A) of different therapeutic options may have underestimated the results concerning whether they would have been analyzed independently. Given the reality of complex treatment combinations for MM and relatively low patient numbers, assessing individual treatment effectiveness will require substantial cohort sizes, which were not possible in our study. Although no differences were found between the groups in baseline characteristics such as sex, disease stage or Charlson comorbidity index, a post-hoc analysis to adjust the PFS and OS data for DVMP and A Group for these characteristics would be recommended to further delve into the survival analysis.

## 5. Conclusions

As in clinical trials, the CARINAE study, conducted in the context of routine clinical practice, shows that adding Daratumumab to the standard three-drug regimen in patients diagnosed with multiple myeloma non-eligible for autologous transplant increases progression-free survival as well as the depth of response, without clinically significant worsening of the tolerance profile, anticipating this benefit even for elderly patients over 80 s.

This highlights the importance of real-world data collection in complementing clinical trial data and provides a broader understanding of patient characteristics and treatment outcomes. Moreover, it provides a glimpse of the impact on patients following the diagnosis, how they live with an incurable disease, and for most of them, how treatment improved their lives.

## Figures and Tables

**Table 1:** Demographic and clinical characteristics at diagnosis.

Characteristics	Total (*n* = 109)	Group A (*n* = 52)	DVMP Group (*n* = 57)	*p* ^1^
Age *median (range)*	75.0 (63.0–88.0)	76.5 (66.0–86.0)	75.0 (63.0–88.0)	0.2393 ^(u)^
Sex *n (%)*				0.7426 ^(c)^
Male	59 (54.1%)	29 (55.8%)	30 (52.6%)	
Female	50 (45.9%)	23 (44.2%)	27 (47.4%)	
Comorbidities *n (%)*				
Cardiopathy	31 (28.4%)	17 (32.7%)	14 (24.6%)	0.3473 ^(c)^
Peripheral neuropathy	3 (2.8%)	3 (5.8%)	0 (0.0%)	0.1053 ^(f)^
Renal failure	27 (24.8%)	16 (30.8%)	11 (19.3%)	0.1658 ^(c)^
Charlson’s comorbidities index median *(range)*	3 (2–4)	3 (2–4)	3 (2–4)	0.3005 ^(u)^
ECOG PS *n (%)*				0.1284 ^(c)^
0	23 (21.1%)	7 (13.5%)	16 (28.1%)	
1	43 (39.4%)	25 (48.1%)	18 (31.6%)	
2	24 (22.0%)	9 (17.3%)	15 (26.3%)	
3	8 (7.3%)	4 (7.7%)	4 (7.0%)	
4	1 (0.9%)	0 (0.0%)	1 (1.8%)	
NA	10 (9.2%)	7 (13.5%)	3 (5.3%)	
Creatinine clearance *n (%)*				0.1980 ^(c)^
<30 mL/min	13 (11.9%)	8 (15.4%)	5 (8.8%)	
30–60 mL/min	32 (29.4%)	18 (34.6%)	14 (24.6%)	
>60 mL/min	52 (47.7%)	23 (44.2%)	29 (50.9%)	
NA	12 (11.0%)	3 (5.8%)	9 (15.8%)	
Myeloma type ^2^ *n (%)*				
IgG ^3^	51 (46.8%)	23 (44.2%)	28 (49.1%)	0.6092 ^(c)^
IgA ^3^	35 (32.1%)	15 (28.8%)	20 (35.1%)	0.4857 ^(c)^
NA	21 (19.3%)	12 (23.1%)	9 (15.8%)	0.3353 ^(c)^
Oligosecretory ^4^	5 (4.6%)	3 (5.8%)	2 (3.5%)	0.6679 ^(f)^
High-risk CG profile (FISH) ^5^ *n (%)*				
*t (4;14) (p16; q32)*	8 (16.0%)	5 (19.2%)	3 (12.5%)	0.7041 ^(f)^
*t (14;16) (q32; q23)*	2 (4.0%)	1 (3.8%)	1 (4.2%)	1.0000 ^(f)^
*Del (17 p)*	5 (10.0%)	2 (7.7%)	3 (12.5%)	0.6613 ^(f)^
No CG abnormalities (FISH) ^6^ *n (%)*	44 (40.4%)	21 (40.4%)	23 (40.4%)	0.4467 ^(c)^
Plasmacytoma ^7,8^ *n (%)*	26 (24.1%)	11 (21.6%)	15 (26.3%)	0.5646 ^(c)^
Lytic bone lesions ^7^ *n (%)*	66 (61.1%)	29 (56.9%)	37 (64.9%)	0.3916 ^(c)^
Disease staging ISS *n (%)*				0.3126 ^(c)^
I	25 (22.9%)	9 (17.3%)	16 (28.1%)	
II	39 (35.8%)	19 (36.5%)	20 (35.1%)	
III	40 (36.7%)	20 (38.5%)	20 (35.1%)	
NA	5 (4.6%)	4 (7.7%)	1 (1.8%)	
Revised disease staging R-ISS *n (%)*				0.3294 ^(c)^
I	19 (17.4%)	6 (11.5%)	13 (22.8%)	
II	54 (49.5%)	28 (53.8%)	26 (45.6%)	
III	17 (15.6%)	10 (19.2%)	7 (12.3%)	
NA	19 (17.4%)	8 (15.4%)	11 (19.3%)	

NA: Not available; CG: Cytogenetic. ^1^ Comparison between treatment groups: Mann-Whitney U test (u), Chi-square (c) test or Fisher’s exact test (f). ^2^ Non-exclusive options. ^3^ *n* = 1 patient with biclonality: both IgG and IgA. ^4^ *n* = 3 patients with Ig also reported but not considered for the analysis. ^5^ Based on *n* = 50 patients with reported cytogenetic abnormalities (*n* = 26 in Group A and *n* = 24 in DVMP Group). ^6^ Based on *n* = 94 patients with reported cytogenetic data (*n* = 47 in Group A and *n* = 47 in DVMP Group). ^7^ *n* = 1 patient without information in lytic bone lesions/plasmacytoma. ^8^ Extramedullary disease was defined as para-skeletal soft tissue masses, soft tissue masses extending beyond the bone marrow, or both.

**Table 2:** Front-line treatment.

Front-Line Treatment ^1^ *n (%)*	Group A (*n* = 52)
**Bortezomib-based regimens**	**30 (57.7%)**
Bortezomib-Melphalan-Prednisone	17 (32.7%)
**Lenalidomide-based regimens**	**14 (26.9%)**
Lenalidomide-Dexamethasone	14 (26.9%)
**Bortezomib + lenalidomide-based regimens**	**4 (7.7%)**
Bortezomib-Lenalidomide-Dexamethasone	3 (5.8%)
**Alkylating Agents-based regimens**	**4 (7.7%)**
Melphalan-Prednisone	2 (3.8%)

^1^ Schemes grouped by therapeutic group. The most frequent therapeutic regimen in each group is shown.

**Table 3:** Progression free survival (PFS).

	Event *n* (%)	Censored *n* (%)	Survival Probability	Log Rank
**Complete follow-up**				0.0524
Group A	37 (71.2%)	15 (28.8%)	0.2306	
DVMP Group	21 (36.8%)	36 (63.2%)	0.5285	
**18-month follow-up**				0.0325
Group A	20 (38.5%)	32 (61.5%)	0.6154	
DVMP Group	11 (19.3%)	46 (80.7%)	0.8070	
**24-month follow-up**				0.0390
Group A	25 (48.1%)	27 (51.9%)	0.5192	
DVMP Group	16 (28.1%)	41 (71.9%)	0.7107	
**36-month follow-up ^1^**				0.0545
Group A	35 (67.3%)	17 (32.7%)	0.3170	
DVMP Group	21 (36.8%)	36 (63.2%)	0.5285	

PFS: Time in months between the date of initiation of first-line treatment and the date of disease progression or death from any cause, whichever comes first. Those patients who did not reach it were censored on the date of the last follow-up. ^1^ *n* = 2 patients (Group A) reported events after 36 months.

**Table 4:** Best response to treatment.

Best Response *n (%)*	Total (*n* = 109)	Group A (*n* = 52)	DVMP Group (*n* = 57)	*p* ^1^
**≥Complete response**	**36 (33.0%)**	**15 (28.8%)**	**21 (36.8%)**	0.3753
**≥Very good partial response**	**73 (67.0%)**	**30 (57.7%)**	**43 (75.4%)**	0.0491
Strict complete response	15 (13.8%)	10 (19.2%)	5 (8.8%)	0.0803
Complete response	21 (19.3%)	5 (9.6%)	16 (28.1%)	
Very good partial response	37 (33.9%)	15 (28.8%)	22 (38.6%)	
Partial response	23 (21.1%)	13 (25.0%)	10 (17.5%)	
Minimum response	3 (2.8%)	2 (3.8%)	1 (1.8%)	
Stable disease	9 (8.3%)	6 (11.5%)	3 (5.3%)	
Disease progression	1 (0.9%)	1 (1.9%)	0 (0.0%)	

^1^ Comparison between treatment groups: Chi-square test.

**Table 5:** First-line treatment progression disease (PD).

Relapse/PD	Total (*n* = 109)	Group A (*n* = 52)	DVMP Group (*n* = 57)	*p* ^1^
First-line treatment PD *n (%)*				0.0004
Yes	44 (40.4%)	30 (57.7%)	14 (24.6%)	
No	65 (59.6%)	22 (42.3%)	43 (75.4%)	
Tye of relapse ^2^ *n (%)*				0.7204
Biological–No treatment	11 (25.0%)	7 (23.3%)	4 (28.6%)	
Biological–Treatment	13 (29.5%)	10 (33.3%)	3 (21.4%)	
Symptomatic relapse	20 (45.5%)	13 (43.3%)	7 (50.0%)	

^1^ Comparison between treatment groups: Chi-square test. ^2^ Based on n = 44 patients with progression disease inf first-line treatment (*n* = 30 in Group A and *n* = 14 in DVMP Group)

**Table 6:** Retrospective follow-up: Adverse reaction to medication (RAMs).

RAMs	Total (*n* = 111)	Group A (*n* = 53)	DVMP Group (*n* = 58)	*p* ^1^
Patients with RAMs *n (%)*				0.4734
Yes	81 (73.0%)	37 (69.8%)	44 (75.9%)	
No	30 (27.0%)	16 (30.2%)	14 (24.1%)	
No RAMs reported	251	129	122	--
Patients with serious RAMs *n (%)*				0.8030
Yes	24 (21.6%)	12 (22.6%)	12 (20.7%)	
No	87 (78.4%)	41 (77.4%)	46 (79.3%)	
No serious RAMs reported	28	13	15	
**RAMs characteristics ^2^**				
Serious *n (%)*				0.5770
Yes	28 (11.2%)	13 (10.1%)	15 (12.3%)	
No	223 (88.8%)	116 (89.9%)	107 (87.7%)	
Severity criterion ^3^ *n (%)*				0.2657
Life-threatening	2 (7.1%)	2 (15.4%)	0 (0.0%)	
Hospitalization	14 (50.0%)	7 (53.8%)	7 (46.7%)	
Hospitalization prolonged	3 (10.7%)	1 (7.7%)	2 (13.3%)	
Other medical reason	8 (28.6%)	2 (15.4%)	6 (40.0%)	
Death	1 (3.6%)	1 (7.7%)	0 (0.0%)	
Intensity *n (%)*				0.0277
Grade 1	104 (41.4%)	48 (37.2%)	56 (45.9%)	
Grade 2	93 (37.1%)	50 (38.8%)	43 (35.2%)	
Grade 3	41 (16.3%)	28 (21.7%)	13 (10.7%)	
Grade 4	11 (4.4%)	2 (1.6%)	9 (7.4%)	
Grade 5	2 (0.8%)	1 (0.8%)	1 (0.8%)	
Result *n (%)*				0.9276
Recovered without sequalae	227 (90.4%)	116 (89.9%)	111 (91.0%)	
Not recovered, improving	7 (2.8%)	4 (3.1%)	3 (2.5%)	
Not recovered, ongoing	9 (3.6%)	4 (3.1%)	5 (4.1%)	
Recovered with sequalae	5 (2.0%)	3 (2.3%)	2 (1.6%)	
Unknown	1 (0.4%)	1 (0.8%)	0 (0.0%)	
Death	2 (0.8%)	1 (0.8%)	1 (0.8%)	

^1^ Comparison between treatment groups: Chi-square test. ^2^ Based on *n* = 251 RAMs (*n* = 129 in Group A and *n* = 122 in DVMP Group). ^3^ Based on *n* = 28 serious RAMs (*n* = 13 in Group A and *n* = 15 in DVMP Group).

**Table 7:** Prospective follow-up: Adverse events (AEs) in first-line treatment.

AEs	Total (*n* = 111)	Group A (*n* = 53)	DVMP Group (*n* = 58)	*p* ^1^
Patients with AEs *n (%)*				<0.0001
Yes	45 (40.5%)	11 (20.8%)	34 (58.6%)	
No	66 (59.5%)	42 (79.2%)	24 (41.4%)	
No of AEs reported	172	39	133	
Patients with serious AEs (SAEs) *n (%)*				0.0002
Yes	17 (15.3%)	1 (1.9%)	16 (27.6%)	
No	94 (84.7%)	52 (98.1%)	42 (72.4%)	
No of SAEs reported	26	3	23	
**AEs characteristics ^2^**				
Serious *n (%)*				0.1411
Yes	26 (15.1%)	3 (7.7%)	23 (17.3%)	
No	146 (84.9%)	36 (92.3%)	110 (82.7%)	
Severity criterion ^3^ *n (%)*				0.6013
Life-threatening	2 (7.7%)	0 (0.0%)	2 (8.7%)	
Hospitalization	20 (76.9%)	2 (66.7%)	18 (78.3%)	
Death	4 (15.4%)	1 (33.3%)	3 (13.0%)	
Intensity *n (%)*				0.3903
Grade 1	86 (50.0%)	23 (59.0%)	63 (47.4%)	
Grade 2	58 (33.7%)	13 (33.3%)	45 (33.8%)	
Grade 3 ^4^	24 (14.0%)	2 (5.1%)	22 (16.5%)	
Grade 4	1 (0.6%)	0 (0.0%)	1 (0.8%)	
Grade 5	3 (1.7%)	1 (2.6%)	2 (1.5%)	
Result *n (%)*				0.3856
Recovered without sequalae	120 (69.8%)	27 (69.2%)	93 (69.9%)	
Not recovered, improving	8 (4.7%)	2 (5.1%)	6 (4.5%)	
Not recovered, ongoing	33 (19.2%)	8 (20.5%)	25 (18.8%)	
Recovered with sequalae	6 (3.5%)	0 (0.0%)	6 (4.5%)	
Unknown	1 (0.6%)	1 (2.6%)	0 (0.0%)	
Death	4 (2.3%)	1 (2.6%)	3 (2.3%)	

^1^ Comparison between treatment groups: Chi-square test. ^2^ Based on *n* = 172 AEs (*n* = 39 in Group A and *n* = 133 in DVMP Group). ^3^ Based on *n* = 26 SAEs (*n* = 3 in Group A and *n* = 23 in DVMP Group). ^4^ *n* = 1 AE grade 3 and classified as severe with initial criteria of death.

## Data Availability

Although these data are not currently publicly available for sharing, requests for sharing can be sent to the Corresponding Author and will be evaluated on an individual basis. The data will be provided after its deidentification in compliance with applicable privacy laws, data protection, and requirements for consent and anonymization.

## Supplementary Materials

The following supporting information can be downloaded at: https://www.mdpi.com/article/10.3390/ph17101272/s1. Table S1. Laboratory parameters; Table S2. First-line treatment characteristics; Table S3. Subsequent treatment characteristics; Table S4. CARINAE Investigators. Figure S1. Progression-free survival censored by other death–disease progression or death due to progression; Figure S2. Patient’s perspective.
