# Supplementary material for: Unravelling Transplant-Ineligible Newly Diagnosed Multiple Myeloma Treatment in Real-World Practice in Spain: The CARINAE Study
Source: Pharmaceuticals (Basel). 2024 Sep 26;17(10):1272. doi: 10.3390/ph17101272 (PMC11510635; doi:10.3390/ph17101272)
Supplement: Supplementary file 1 [file pharmaceuticals-17-01272-s001.zip › pharmaceuticals-3149165-supplementary.pdf]

Table S1. Laboratory parameters

Parameters	Total (n=109)	Group A (n=52)	DVMP Group (n=57)	p[1]
Hemoglobin (g/dL)				
Mean (SD)	11.2 (2.2)	10.8 (2.3)	11.6 (2.1)	0.0514[(t)]
Median (P25 – P75)	11.1 (9.6 ; 12.6)	10.4 (9.3 ; 12.4)	11.4 (10.2 ; 13.0)	
Valid n	*109*	*52*	*57*	
Calcium (mg/dL)				
Mean (SD)	9.6 (0.9)	9.6 (1.1)	9.6 (0.7)	0.1305[(u)]
Median (P25 – P75)	9.4 (9.1 ; 9.9)	9.4 (9.0 ; 9.8)	9.5 (9.3 ; 10.0)	
Valid n	*108*	*52*	*56*	
Albumin (g/dL)				
Mean (SD)	3.7 (0.6)	3.7 (0.7)	3.8 (0.5)	0.3718[(t)]
Median (P25 – P75)	3.8 (3.3 ; 4.1)	3.7 (3.2 ; 4.2)	3.8 (3.4 ; 4.1)	
Valid n	*105*	*52*	*53*	
β-2 microglobulin (ml/L)				
Mean (SD)	6.3 (5.6)	6.7 (5.8)	5.8 (5.3)	0.4140[(u)]
Median (P25 – P75)	4.4 (3.1 ; 7.3)	4.4 (3.6 ; 7.6)	4.4 (3.1 ; 6.5)	
Valid n	*100*	*50*	*50*	
LDH *n (%)*				0.8199[(c)]
Normal	92 (86.8%)	43 (86.0%)	49 (87.5%)	
Increased	14 (13.2%)	7 (14.0%)	7 (12.5%)	
M protein in serum (mg/dL)				
Mean (SD)	2751.0 (2901.3)	2502.7 (2037.3)	2972.3 (3501.5)	0.6936[(u)]
Median (P25 – P75)	2430.0 (1108.5 ; 3615.0)	2460.0 (460.0 ; 3480.0)	2410.0 (1480.0 ; 3759.0)	
Valid n	*104*	*49*	*55*	
M protein in urine (mg/24h)				
Mean (SD)	662.8 (1053.4)	670.5 (1159.0)	655.1 (953.7)	0.4127[(u)]
Median (P25 – P75)	120.0 (3.0 ; 951.2)	98.5 (0.0 ; 630.5)	141.2 (8.5 ; 980.0)	
Valid n	*68*	*34*	*34*	
Kappa (κ) FLC in serum (mg/dL)				
Mean (SD)	181.4 (836.7)	308.7 (1182.9)	59.0 (99.7)	0.8888[(u)]
Median (P25 – P75)	18.0 (1.3 ; 81.6)	17.8 (1.3 ; 117.0)	18.8 (1.3 ; 80.4)	
Valid n	*104*	*51*	*53*	
Lambda λ FLC in serum (mg/dL)				
Mean (SD)	267.3 (1167.8)	285.5 (1117.5)	250.1 (1223.6)	0.7558[(u)]
Median (P25 – P75)	2.6 (0.7 ; 36.1)	2.8 (0.7 ; 41.8)	1.7 (0.7 ; 36.1)	
Valid n	*105*	*51*	*54*	
FLC κ and λ ratio				
Mean (SD)	144.9 (394.5)	175.1 (418.3)	115.8 (372.0)	0.7708[(u)]
Median (P25 – P75)	6.5 (0.0 ; 60.0)	6.9 (0.0 ; 87.5)	2.3 (0.0 ; 54.6)	

Parameters	Total (n=109)	Group A (n=52)	DVMP Group (n=57)	p[1]
Valid n	*102*	*50*	*52*	

SD – standard deviation; *95% IC: 95% confidence interval;* P25 – 25th percentile; P75 – 75th percentile; min – minimum; max - maximum

[1] Comparison between treatment groups: Student t-test (t), Mann-Whitney U test (u), Chi-square test (c)

Table S2. First-line treatment characteristics

Characteristics	Total (n=109)	Group A (n=52)	DVMP Group (n=57)	p[1]
Treatment until progression *n (%)*				<0.0001(c)
Yes	96 (88.1%)	39 (75.0%)	57 (100.0%)	
No	13 (11.9%)	13 (25.0%)	0 (0.0%)	
**Treatment until progression[2]**				
Treatment interruption *n (%)*				0.0001(c)
Yes	51 (53.1%)	30 (76.9%)	21 (36.8%)	
No	45 (46.9%)	9 (23.1%)	36 (63.2%)	
Treatment days until interruption[3]				
Mean (SD)	365.7 (306.5)	384.9 (343.4)	338.3 (250.1)	0.9466(u)
Cycles received				
Mean (SD)	18.2 (11.7)	17.9 (14.6)	18.4 (9.5)	0.6428(u)
Interruption reason[3] *n (%)*				0.1215(c)
Patient decision	1 (2.0%)	0 (0.0%)	1 (4.8%)	
Related adverse events	16 (31.4%)	12 (40.0%)	4 (19.0%)	
Medical decision for other reasons	27 (52.9%)	16 (53.3%)	11 (52.4%)	
Progression[4]	14 (51,9%)	9 (56,3%)	5 (45,5%)	
Los to follow-up	0 (0.0%)	0 (0.0%)	0 (0.0%)	
Death	7 (13.7%)	2 (6.7%)	5 (23.8%)	
**Fixed treatment[5]**				
Treatment interruption[6] *n (%)*				--
Yes	12 (92.3%)	12 (92.3%)	--	
No	1 (7.7%)	1 (7.7%)	--	
Treatment days until interruption[6]				
Mean (SD)	335.5 (123.8)	335.5 (123.8)	--	--
Cycles received				
Mean (SD)	8.5 (2.2)	8.5 (2.2)	--	--
Interruption reason[6] *n (%)*				--
Treatment completion	7 (58.3%)	7 (58.3%)	--	
Patient decision	1 (8.3%)	1 (8.3%)	--	
Related adverse events	1 (8.3%)	1 (8.3%)	--	
Medical decision for other reasons	3 (25.0%)	3 (25.0%)	--	

[1] *Comparison between treatment groups: Mann-Whitney U test (u), Chi-square test ©*

[2] *Based on n=96 patients with treatment until progression (n=39 in Group A and n=57 in DVMP Group)*

[3] *Based on n=51 patients with treatment until progression and treatment interruption (n=30 in Group A and n=21 in DVMP Group)*

[4] *Based on n=27 patients with treatment until progression and interruption due to medical decision for other reasons (n=16 in Group A and n=11 in DVMP Group)*

[5] *Based on n=13 patients with finite treatment (n=13 in Group A and n=0 in DVMP Group)*

[6] *Based on n=12 patients with finite treatment and treatment interruption (n=12 in Group A and n=0 in DVMP Group)*

Table S3. Subsequent treatment characteristics

Characteristics	Total (n=109)	Group A (n=52)	DVMP Group (n=57)	p[1]
Subsequent multiple myeloma treatments during follow-up *n (%)*				<0.0001
Yes	44 (40.4%)	31 (59.6%)	13 (22.8%)	
No	65 (59.6%)	21 (40.4%)	44 (77.2%)	
Time to the next anti-myeloma treatment (years)[2]				
Mean (SD)	2.1 (1.0)	2.3 (1.2)	2.0 (0.7)	0.0701
Anti-CD38 treatment[3] *n (%)*				0.2817
Yes	19 (43.2%)	15 (48.4%)	4 (30.8%)	
No	25 (56.8%)	16 (51.6%)	9 (69.2%)	

[1] *Comparison between treatment groups: Chi-square test*

[2] *Years elapsed to the next anti-myeloma treatment (or date of last follow-up):*

- *Patients with the next anti-myeloma treatment after progression of first-line treatment:*
  (Date of next anti-myeloma treatment after progression - Date of start of first-line treatment) / 365.25
- *Patients who died from any cause or had no anti-myeloma treatment due to progression:*
  (Date of last follow-up - Date of start of first-line treatment) / 365.25

[3] *Based on n=44 patients with subsequent multiple myeloma treatment (n=31 in Group A and n=13 in DVMP Group)*

Table S4. CARINAE investigators

Investigator	Site
Abelardo Bárez García	Hospital de Ávila
Alberto Marín Sánchez	Hospital General Universitario de Albacete
Alfonso García de Coca	Hospital Central Universitario de Valladolid
Ana Dios Loureiro	Complejo Hospitalario Universitario de Pontevedra (CHUP)
Ana López de la Guía	Hospital Universitario La Paz
Antonia Sampol Mayol	Hospital Universitario Son Espases
Antoni Garcia Guiñón	Hospital Universitario Arnau de Vilanova de Lleida
Carmen Martínez Chamorro	Hospital Universitario Quirónsalud Madrid
Cristina Motlló Borrella	Hospital de Sant Joan de Déu
Enrique M. Ocio San Miguel	Hospital Universitario Marqués de Valdecilla
Esther Clavero Sánchez	Hospital Universitario Virgen de las Nieves
Eugenia Abella Monreal	Hospital del Mar (Barcelona)
Eugenio Giménez Mesa	Hospital Universitario Infanta Sofía
Felipe De Arriba De la Fuente	Hospital General Universitario Morales Meseguer
Juan Alfonso Soler Campos	Hospital de Sabadell Parc Taulí
Joan José Bargay Lleonart	Hospital Universitario Son Llàtzer
Judith Vázquez Álvarez	Hospital Álvaro Cunqueiro
Mª José Moreno Belmonte	Hospital Central Universitario Virgen de la Arrixaca
María Ángeles Andreu Costa	Hospital Universitario de Móstoles
María Casanova Espinosa	Hospital Costa del Sol (IVCS)
María Magdalena Alcalá Peña	Hospital Universitario Regional de Málaga
Marta Romera Martínez	Hospital General Universitario Santa Lucía
Marta Sonia González Pérez	Complejo Hospitalario Universitario de Santiago (CHUS)
Mercedes Gironella Mesa	Hospital Universitario Vall d'Hebrón
Miguel Teodoro Hernández García	Hospital Universitario de Canarias
Rafael Duro Millán	Hospital Universitario Virgen Macarena
Ricarda García Sánchez	Hospital Universitario Virgen de la Victoria
Susana Herráez Rodríguez	Hospital Universitario Basurto
Vicente Carrasco Baraja	Hospital Royo Villanova
Virginia Cardos Gómez	Hospital Nuestra Señora del Prado

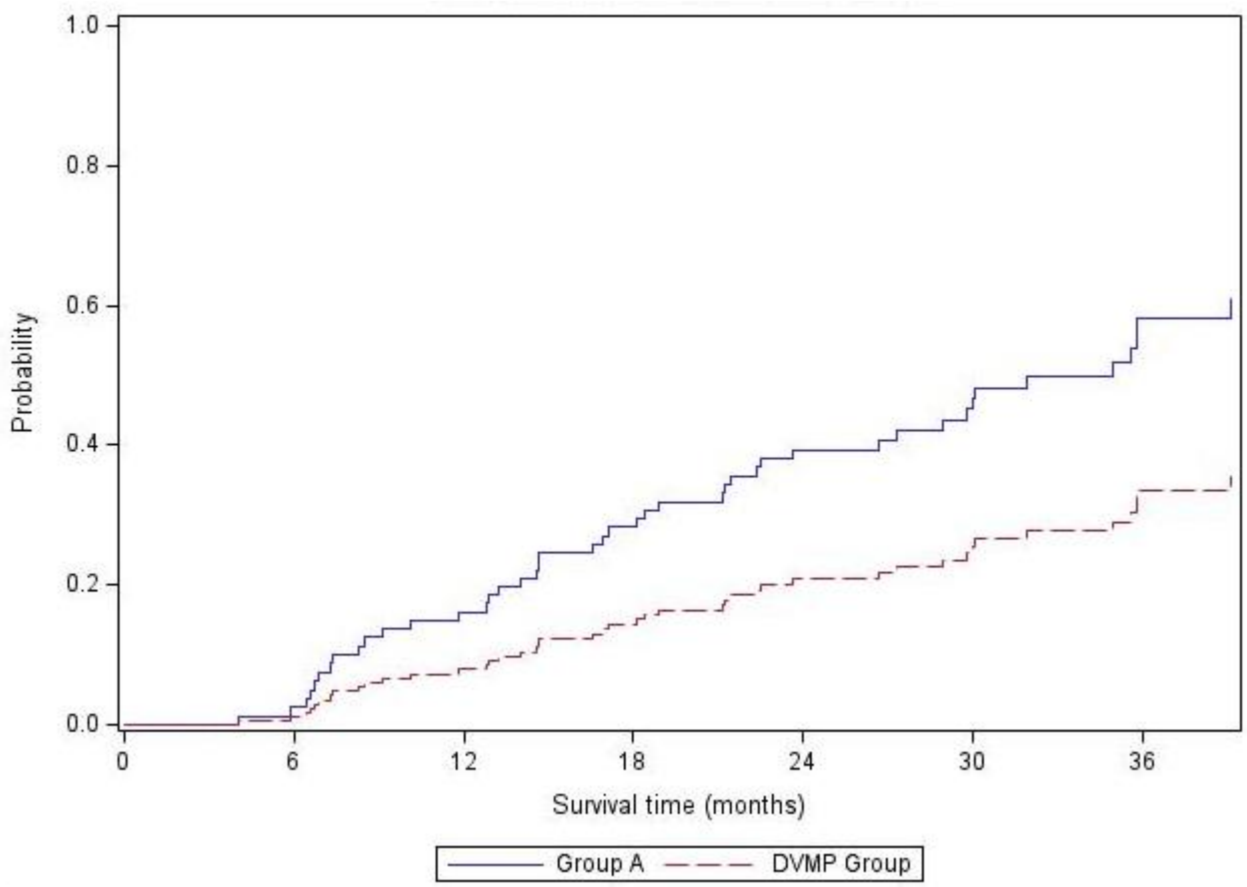

Figure S1. Progression-free survival censored by other death -disease progression or death due to progression.

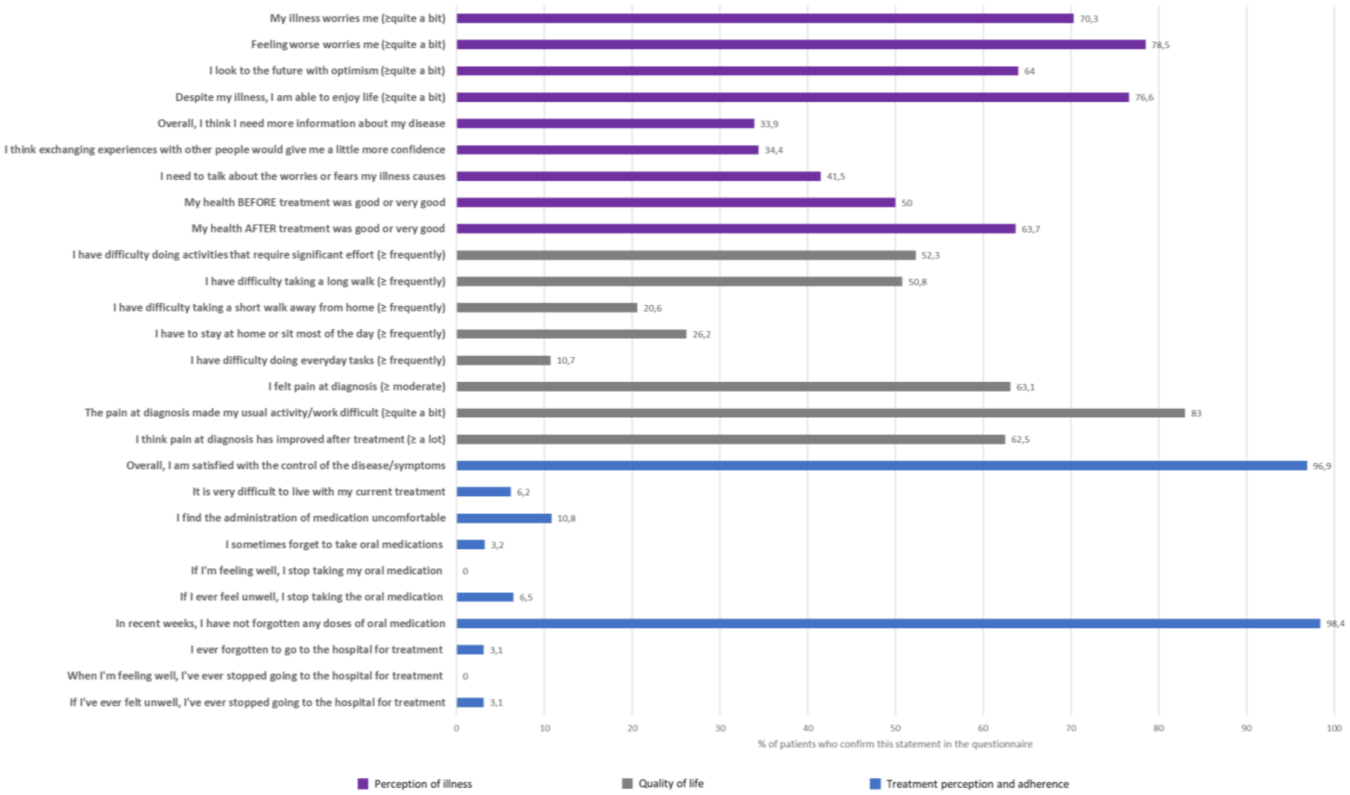

Figure S2. Patient's perspective
